# Tailocin as an alternative weapon to type VI secretion system in bee gut symbionts

**DOI:** 10.64898/2026.10.28.762571

**Published:** 2026-10-29

**Authors:** Yulin Song, Nazhin Nikaeen, Nurila Kambar, Korin R. Jones, Madison Wade, Cecilia Leal, Nancy A. Moran

## Abstract

Antagonistic interactions often shape microbial communities, as co-residents compete for resources. One system of interbacterial antagonism involves tailocins: phage tail-like particles produced by some bacteria to kill close relatives. Their roles in natural communities are poorly studied. Here, we identified putative tailocin loci ubiquitously present in genomes of *Snodgrassella* species, core members of the bee gut microbiota. We purified tailocins from culture supernatants of different *Snodgrassella* strains and found that these inhibited growth of multiple *Snodgrassella* strains but not of strains of other species that cooccur in bee guts. Proteomic analyses corroborated the production of tailocins, and cryogenic electron microscopy imaging revealed their morphology and killing activity via membrane disruption. We generated single- and double-gene knockout mutants of a predator *Snodgrassella* strain that naturally possesses both tailocin and a type VI secretion system (T6SS) that was previously shown to function in intraspecies competition in bee guts. These mutants were used in tests for antagonism against a prey *Snodgrassella* strain in vivo. Co-inoculations within gnotobiotic honeybees including visualization within guts showed that both tailocin and T6SS are important for *Snodgrassella* in intraspecies competition. Each of these two antagonism mechanisms inhibits the establishment of susceptible strains within the ileum. Outcomes depend on the timing of colonization by competing strains, with both tailocin and T6SS giving a stronger advantage when the producing strain colonizes first. Thus, tailocin production can underlie priority effects that have been widely observed in the bee gut community and other host-associated bacterial communities.

**Significance Statement:**

Interbacterial competition plays a pivotal role in determining bacterial colonization success in natural habitats, including guts of animals. Tailocins are phage tail-like bacteriocins that are widely encoded in bacterial genomes and that function in intraspecies killing. They have been little studied in natural communities, including those in gut environments. Here, we show that bee gut bacteria produce tailocins that kill close relatives within the gut. Tailocins function as phage-derived antagonism mechanisms together with T6SS, which are also phage-derived. The possession of both weapons provides a double safeguard, supporting that intense competition happens in bee gut environments. Our findings bring new insights into roles of antagonistic weapons in natural communities.

## Full Text Availability

The license terms selected by the author(s) for this preprint version do not permit archiving in PMC. The full text is available from the preprint server.

